# Native Sheep Breeds in Poland—Importance and Outcomes of Genetic Resources Protection Programmes

**DOI:** 10.3390/ani12121510

**Published:** 2022-06-09

**Authors:** Aldona Kawęcka, Marta Pasternak, Anna Miksza-Cybulska, Michał Puchała

**Affiliations:** Department of Sheep and Goat Breeding, National Research Institute of Animal Production, 32-083 Balice, Poland; marta.pasternak@iz.edu.pl (M.P.); anna.miksza@iz.edu.pl (A.M.-C.); michal.puchala@iz.edu.pl (M.P.)

**Keywords:** Polish native sheep breeds, genetic resources, protection, programs

## Abstract

**Simple Summary:**

The aim of this paper was to present the current situation of native sheep breeding in Poland, in terms of the significance and the effects of genetic resources protection programmes. The sheep farming tradition in Poland is deeply rooted in culture, particularly in the mountain and foothill regions. Sheep are permanently linked to many areas of country, not only playing an indispensable part in shaping the landscape, but also providing unique products. The most effective method of conserving native breeds is their sustainable management. It is important to maintain and develop the promotion and certification of high-quality products from native breeds and to control the endangered status of local breeds, which must be monitored and updated on an ongoing basis.

**Abstract:**

The sheep population of native breeds, despite their unique features and the ability to adapt to harsh environmental conditions, has significantly decreased in recent years. Due to the low profitability of breeding, many local breeds of sheep in Poland were exposed to the risk of extinction. Many years of crisis in sheep farming have exacerbated this situation. The aim of this paper was to present the current situation of native sheep breeding in Poland, in terms of significance and effects of genetic resources protection programmes. The conservation of genetic resources of sheep aims to maintain and increase the population size while striving to maintain the greatest possible genetic variability. There are 17 native breeds included in the Polish sheep genetic resources conservation programme. A positive element of the implementation of the conservation of genetic resources programme for sheep is the accompanying measures which are based on the use of the non-productive role of the species. Extensive sheep grazing, as a form of nature conservation, serves to preserve valuable natural landscapes and the culture of local communities associated with sheep farming. Production and promotion of quality products, especially using niche markets and short production chains, are essential to ensure the economic viability of farms. These activities must be accompanied by raising public awareness of indigenous breeds and their alternative use in environmental activities, as well as their role in preserving the cultural heritage of local communities, for example through mountain grazing and the production of traditional products.

## 1. Introduction

Native breeds of sheep, despite their unique characteristics and ability to adapt to difficult environmental conditions, have declined in number, and this decline exposes them to the threat of extinction due to the low profitability of breeding. This fate has befallen 5 local breeds of sheep in Poland, and years of crisis in sheep farming have exacerbated this situation. Today, the significance of native breeds is growing, due to their role in the history of development of the regions from which they originate. Through fulfilling important natural, landscape, as well as social and cultural functions, native breeds are a testimony to the traditions and material culture of local communities. Programmes for the conservation of farm animal genetic resources implemented in many countries make it possible to preserve native breeds for future generations. The aim of this paper was to present the current situation of native sheep breeding in Poland, in terms of the significance and the effects of genetic resources protection programmes.

## 2. Global Sheep Genetic Resources

Domestic sheep (*Ovis aries*) are one of the most numerous and earliest domesticated animal species. Since the beginning of domestication, which probably dates back approximately 11,000–9000 years ago in central or Southwestern Asia [1], sheep have accompanied humans in their migrations and colonisation of successive areas. Due to their versatility of use, their importance grew. As the animals moved, they adapted to new conditions. Distinct types of sheep began to develop in terms of physical and physiological characteristics, resulting in breeds that later became an effect of the conscious actions of breeders. Sheep are the species of greatest breed diversity of livestock; according to the World Watch List, there are 1400 breeds worldwide [2]. In 2018, 1520 sheep breeds were recorded, with the Europe and the Caucasus regions being the richest in this respect, with more than half of all known sheep breeds, while North America is the least diverse [3]. Local sheep breeds account for 80% of all sheep breeds worldwide and over 200 sheep breeds are recognised as transboundary, of which more than half are regional breeds. An example of international transboundary breeds is the Suffolk meat sheep found in as many as 46 countries, Texel in 32, Caraculas in 25, and Awassi and East Friesian dairy sheep in 20 countries [4].

As part of the classification elaborated in the Food and Agriculture Organization of the United Nations (FAO) databases and information system, Domestic Animal Diversity Information System (DAD-IS) [5], the division of breeds by origin was adopted. “Native breeds” is a term adopted only in the European region, which means a breed originating in a given country, produced from genetic material available at the time of its creation and adapted to one or more traditional systems of maintenance. The same native breed may be recognised in several countries. The classification also shows imported breeds—foreign to a given country and locally adapted breeds—that have been present in a country long enough to be genetically adapted to one or more production systems or environments of that country [6].

## 3. Programmes for the Conservation of Genetic Resources of Farm Animals in Poland

In 1996 Poland joined the group of implementers of the World Conservation Strategy for Animal Genetic Resources undertaken by FAO, as a result of which it was obliged to establish appropriate national structures. A National Coordination Centre for the Preservation of Animal Genetic Resources was established which, along with the authorisation to collect and store biological material subject to cryopreservation, was transferred to the National Research Institute of Animal Production (NRIAP) [7]. The advisory team and working teams for the conservation of genetic resources of individual animal species, such as cattle, horses, sheep and goats, swine, poultry, fur animals, fish, and bees, are based at the Centre. The Coordination Centre cooperates with the Ministry of Agriculture, the Ministry of Environment, research and educational institutes, non-governmental organisations, and breeders [7,8].

In 1999, based on the working team activities, efforts were undertaken on the National Programme for the Protection of Animal Genetic Resources. As a result, by the decision of the Minister of Agriculture and Rural Development, the implementation of breeding programmes for the conservation of genetic resources of farm animal populations was undertaken. At that time, 32 programmes for the conservation of genetic resources were approved, covering 75 breeds, varieties, lines, and lineages of farm animals, including fish. The programmes specified objectives and schedules of activities, as well as the scope of in-situ and ex-situ conservation (they defined the necessities and the scope of collection and storage of biological material in ex-situ gene banks and the principles for the use of this material). As part of national programmes, a gene bank is envisaged for cattle, sheep, fish, and horses. The programmes also include methods of breeding and indicate the bodies responsible for their implementation. They are subject to amendment and extension when necessary [7,9].

In June 2001, the Minister of Agriculture and Rural Development declared Poland’s participation in the process initiated by FAO to prepare a Report on the State of Animal Genetic Resources in the World. The definitive version of the Report, which emerged after numerous discussions during the National Workshop and the meetings of the Consultative Team, was drafted at the NRIAP and the Report was approved by the Ministry in August 2002 [9].

## 4. Polish Native Sheep Breeds

The sheep farming tradition in Poland is deeply rooted in culture, particularly in the mountain and foothill regions. Sheep are permanently linked to many areas in Poland, not only playing an indispensable part in shaping the landscape, but also providing unique products such as wool, leather, meat, and dairy products [10]. The protection of sheep genetic resources has been conducted in our country since the 1970s. Owing to the initiative of the NRIAP, the Wrzosówka sheep, considered to be an extinct breed, was restored, and reintroduced to breeding through restitution activities. The Institute bought out the Wrzosówka from peasant farms and established their breeding in experimental plants. A comparable situation applied to other breeds (Świniarka and Olkuska sheep) whereby a group of enthusiasts working in sheep-breeding unions and agricultural institutes contributed to the success of these activities [8].

**Figure 1:**
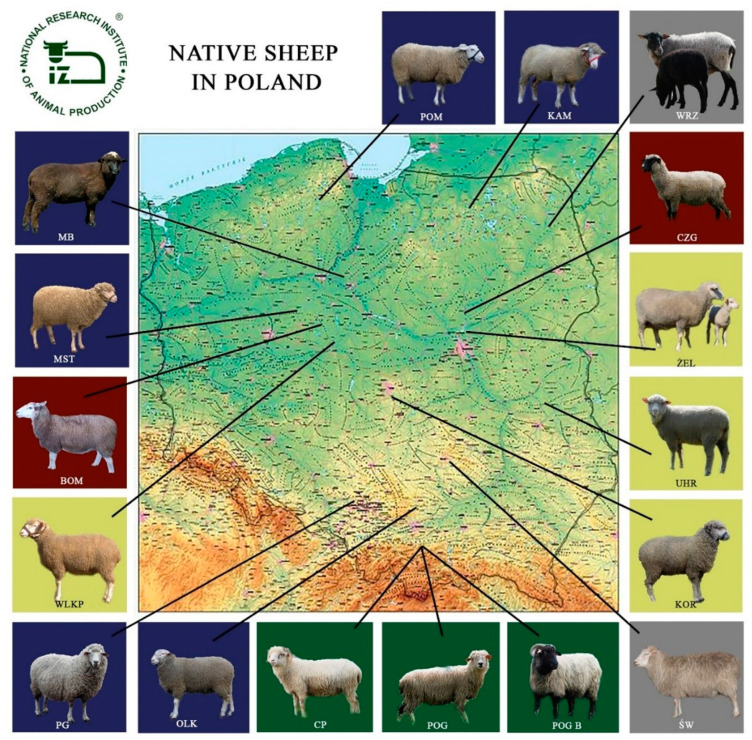
Main regions of origin and occurrence of native breeds of sheep in Poland—Pomeranian (POM), Kamieniecka (KAM), Wrzosówka (WRZ), Black-headed (CZG), Żelaźnieńska (ŻEL), Uhruska (UHR), Corriedale (KOR), Świniarka (ŚW), Coloured Mountain Sheep (POG B), Polish Mountain Sheep (POG), Podhale Zackel (CP), Olkuska (OLK), Polish Pogórza sheep (PG), Wielkopolska (WLKP), White-headed meat sheep (BOM), Old-type Polish Merino (MST), Coloured Merino (MB).

In the 1990s, there was a drastic reduction in the overall sheep population in Poland, and thus many breeds that were less productive were at risk of being eliminated from breeding. The programme for the protection of genetic resources of sheep, as well as the farm and environment programmes of the Rural Development Programme, implemented since 2004, have made it possible to protect breeds in danger of extinction. In 1999, the Programme for the Protection of Genetic Resources included twelve sheep breeds such as Świniarka and Wrzosówka, which have been bred in Poland for centuries, as well as the following breeds: Olkuska, Pomorska, Coloured Merino, Uhruska, Wielkopolska, Corriedale, Żelaźnieńska, and Kamieniecka. Due to the low interest of breeders, the protection of the Leine and Booroola breeds was abandoned during the implementation of the programs. In 2000, coloured mountain sheep originated from the Zackel sheep were added to the programme. In 2008, the old-type Polish Merino and the Podhale Zackel were also incorporated into the programme. In 2015, Polish foothill sheep and the Black-headed sheep became part of the programme [11,12]. As of 2022, there are seventeen breeds included in the sheep genetic resources conservation programme. Figure 1 shows the main regions of origin and occurrence of local sheep breeds; Table 1 shows their characteristics.

The native breeds are adapted to often difficult and demanding environmental conditions of the regions from which they originate. They are distinguished by lower productivity than high-performance and commercial breeds, however, it is compensated by their resistance to diseases and stress, high fertility and prolificacy, longevity and low nutritional requirements with good use of feed [8].

## 5. Financial Support for Actions Related to the Conservation of Genetic Resources

The implementation of conservation programmes requires the provision of financial support, since populations of native breeds are distinguished by lower productivity and, consequently, lower profitability of breeding in relation to breeds used in intensive production. Prior to Poland’s accession to the European Union (EU), financial support for the implementation of projects resulting from the maintenance of local breeds in accordance with adopted conservation programmes was covered under the national budget. Since 2005, funding for activities related to the protection of animal genetic resources has come from two sources and provided directly to the breeders. Support for breeders of horses, cattle, sheep, goats, and pigs is covered by farm and environment payments as part of the Rural Development Programme (to a considerable extent these are EU funds). Breeders of poultry, fur animals, bees, and fish covered by the genetic resources protection programme benefit from national aid [7,13].

Since 2004, breeders who keep sheep, horses, cattle, pigs, and since 2015, those who keep goats covered by conservation programmes, have benefited from EU funds (the farm and environment programmes of the Rural Development Programme 2004–2006, 2007–2013, and 2014–2020). The number of subsidies for animals of conservation breeds has been estimated to calculate the opportunity costs in these payments. Package 7 under the farm, environment, and climate programme of Rural Development Programme 2014–2020 supports programmes for the conservation of animal genetic resources, including sheep and goats. The aim of the package is to financially support the protection of particularly valuable breeds of selected farm animal species in case of which a low or decreasing number of breeding individuals results in the risk of their extinction (https://www.gov.pl/web/arimr/informacje---pakiet-7-zachowanie-zagrozonych-zasobow-genetycznych-zwierzat-w-rolnictwie, accessed on 1 March 2022). The NRIAP, which is authorised to implement or coordinate activities in the field of genetic resources protection, defines criteria and abundance thresholds at which a given breed becomes endangered, creates a protection programme the implementation of which is to ensure the protection of individual endangered breeds, supervises the implementation, and coordinates genetic resources protection programmes [7,12].

Farm, environmental, and climate payments are granted for ewes, which, depending on the breed, range from 10 to 30 animals per flock. The general conditions for participation in the programme for the conservation of genetic resources of sheep, hereafter referred to as the programme, are set out in a procedure available at www.bioroznorodnosc.izoo.krakow.pl (accessed on 1 March 2022). Participation in the conservation programme is voluntary. The payment under this package is granted to a recipient who owns a farm located in Poland, with arable land of not less than 1 ha. The payments under the measure are granted annually, for the period of 5-year commitment, to farmers who voluntarily accept the farm, environmental, and climate obligation within the scope of the package or variant concerned. The payment fully or partially compensates for lost income and additional costs incurred. Currently, The farm, environment, and climate payment under the package “Conservation of local sheep breeds” amounts to around €100 per ewe per year [12].

## 6. Activities Performed as Part of the Coordination of Protection Programmes

**Figure 2:**
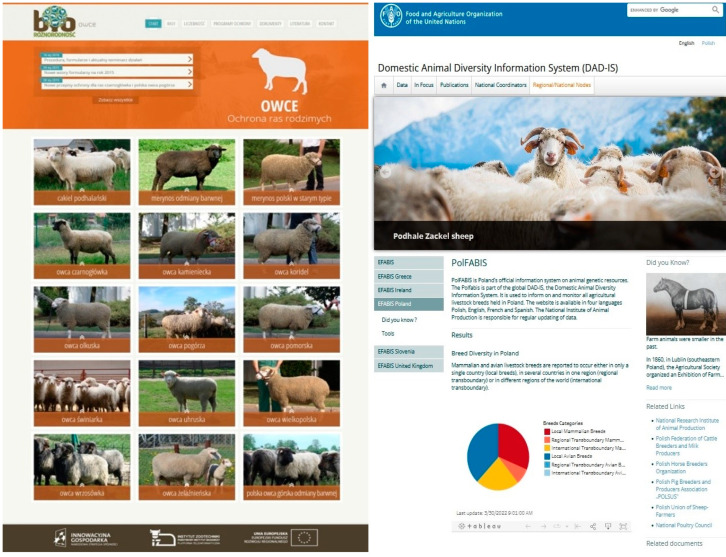
NRIAP biodiversity website-native breeds of sheep in Poland (http://owce.bioroznorodnosc.izoo.krakow.pl/, accessed on 1 March 2022) and Polish EFABIS website (https://www.fao.org/dad-is/regional-national-nodes/efabis-pol/en/, accessed on 1 March 2022).

The NRIAP in Cracow performs and coordinates tasks related to the protection of genetic resources (Journal of Laws 2021, item 36) [14], assigned by the Minister of Agriculture and Rural Development under the Act of 10 December 2020, on the organisation of animal breeding and reproduction of animals [14]. The Institute oversees all work related to the protection of livestock genetic resources, and these activities include several key issues such as:

- selection of sheep breeds for the conservation programme when it is necessary to include an endangered breed
- selection for the purpose of the conservation programme of animals and flocks when new breeds are introduced
- drawing up complete documentation necessary for the implementation of measures under the programme, procedures, sample documents to be submitted by recipients under farm and environment programmes
- maintenance and management of a database on national genetic resources of sheep
- maintaining a website devoted to the implementation of sheep and goat genetic resources protection programmes (Figure 2)
- updating data on Polish sheep and goat breeding in the European Farm Animal Biodiversity Information System (EFABIS) (Figure 2)
- cooperation with FAO and other international organisations on the implementation of the World Action Plan for Animal Genetic Resources
- activities related to the popularisation of native sheep breeds through publication of scientific research results on native sheep breeds, organisation of national and regional exhibitions of native animal breeds, symposia, trainings, and scientific conferences.
- research: the currently conducted scientific research concerns the characteristics of the quality of products from native breeds of sheep in terms of nutritional and health-promoting values, their use in maintaining the biodiversity of naturally valuable areas, as well as the assessment of genetic variability of the population with the use of modern molecular biology tools
- projects: the research project “BIOSTRATEG—The directions of use and the conservation of farm animal genetic resources under sustainable development” carried out in the recent period at the NRIAP. It was an interdisciplinary concept of comprehensive research related to the protection and the use of the potential of native animal breeds, including sheep, in low-input production systems, paying particular attention to the dietary and health-promoting properties of products and the development of naturally valuable areas. The impact of animal, biotechnological, and economic sciences on the socio-economic and natural environment by enabling the use of the latest technologies and solutions for the protection of animal genetic resources in order to optimally use their production potential

## 7. Results of Sheep Protection Programmes Implementation

### 7.1. Increase in Population Size

**Figure 3:**
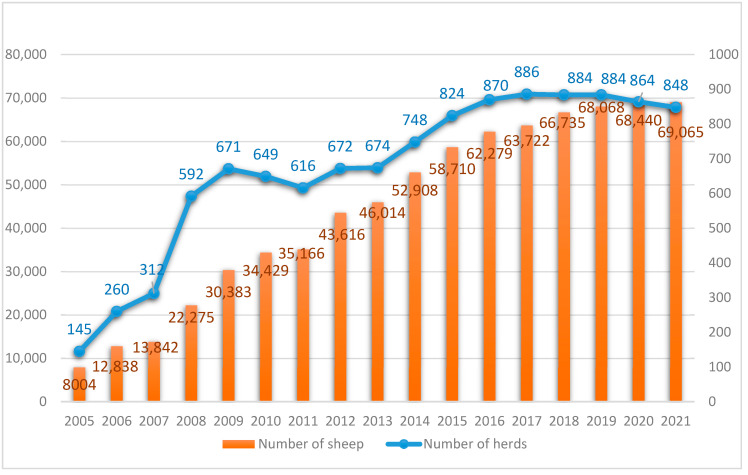
Changes in the size of the protected population of all native breeds of sheep in Poland.

The programme for the conservation of genetic resources in sheep has been a useful tool to protect the biodiversity of this species. Since the beginning of the programme for the conservation of sheep genetic resources, there has been a steady increase in the protected population. Within 15 years of the implementation of the programme (Figure 3), the number of sheep increased over eightfold: from eight thousand ewes in 2005 to over sixty-nine thousand in 2021, kept in 848 flocks all over Poland. Since 2008 and the inclusion of the Old-type Polish Merino Sheep in the conservation programme along with the Podhale Zackel, there has been a significant, almost twofold increase in the number of flocks in comparison to the number recorded in the previous year. This was due to the more numerous initial populations of these two sheep breeds. In 2015, two other breeds were included in the conservation programme: Polish Pogórza sheep and Black-headed sheep. With much smaller populations, therefore, the increase in their numbers was not so rapid. The dynamics of activities related to the implementation of protection programmes have had and still have an impact on sheep breeding in Poland. Due to the possibility of obtaining subsidies for animals covered by the protection programme, breeders are more willing to keep native breeds, for which subsidies were provided, than other breeds of sheep. The native breeds of sheep continue to expand, but these changes are occurring within existing herds. Therefore, the number of herds has started to stabilize in recent years. In 2005, ewes covered by the genetic resources protection programme made up 8% of the total population of ewes entered in the books, while in 2020 they accounted for 80% [11,15,16].

### 7.2. Associated Actions

A positive element in the implementation of the programme for the conservation of genetic resources of sheep is the accompanying measures that are based on the use of the non-productive role of this species. Extensive sheep grazing, as a form of nature conservation, serves to preserve valuable natural landscapes and the culture of local communities associated with sheep farming.

#### 7.2.1. Ecosystem Services

The native breeds of sheep are adapted to extensive grazing in nature-value areas and areas under nature conservation intended to control vegetation and maintain the landscape. This is carried out on wet meadows, fallow land, grasslands in mountain and foothill areas, in river valleys and forest clearings where, after abandoning grazing and haymaking, overgrowth has been observed along with consequent damage to the plants and animal species. Grazing helps preserve the specific nature of ecosystems and biodiversity of free-living species [17]. Grazing is used as a management tool within the protected areas of most national parks in Poland.

Easement grazing, understood as limited collective grazing of sheep and comprehensive pastoral management, is a special form of farming carried out in the mountains, in legally protected areas or in their immediate vicinity. In Poland, easement grazing is practised in all national mountain parks, where it is only permitted in accordance with a number of restrictions. Only shepherds who obtain a permit and sign a lease agreement with the national park authorities may graze sheep. The density of the sheep must be appropriate to the land area, and only native breeds of sheep, mainly mountain sheep (Podhale Zackel, Polish Mountain Sheep, Coloured Mountain Sheep), may be grazed. Shepherds are obliged to observe traditional rituals, cheese-making rules, use traditional equipment, wear traditional clothes, which allow them to cultivate the heritage of the region [17].

#### 7.2.2. Culture-Forming Functions

**Figure 4:**
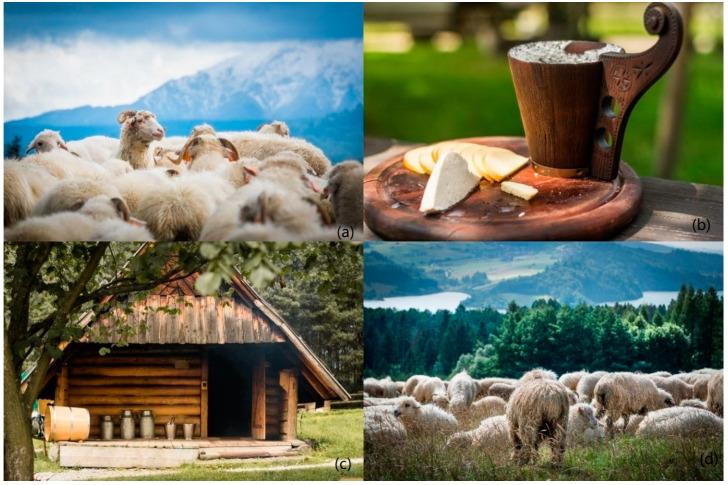
Cultural grazing of mountain sheep in the Polish Carpathians: (**a**) large-scale grazing of native mountain breeds of sheep, (**b**) traditional products made of Polish mountain sheep milk, (**c**) shepherd’s hut, (**d**) large-scale grazing of native mountain breeds of sheep (Photography. M. Pasternak).

Grazing animals in mountain areas fulfils a specific cultural function that distinguishes these areas from other parts of the country, giving a specific character to the entire highland culture. The shepherding events that accompany sheep farming, the production of sheep’s milk products, the cultivation of traditions in shepherd’s huts and sheep grazing by the trails are an unusual attraction for tourists visiting the Polish mountains (Figure 4). Shepherding is of economic and social importance. Through stimulation of tourism, it contributes to the creation of new jobs and improves the welfare of local people. Therefore, there is a need to formalize cultural grazing by developing a social programme considering many aspects of preserving this form of management, in terms of its protection as a culture-creating factor, significantly affecting the development of the equity of local communities [17].

#### 7.2.3. Development of the Traditional Products Market

A critical issue related to conservation breeding is the development of a market for traditional and regional products related to local sheep breeds [12]. Products derived from native breeds of sheep, goats, and cows, kept on mountain pastures with a diverse botanical composition of sward rich in herbs, are fully in line with the current needs of modern consumers who are looking for food that is not only tasty but also healthy. In Western European countries, one can find dairy products (mainly cheese) and cured meat products originating from specific breeds. In Poland, products from native breeds are also available. Mountain sheep are the only dairy-producing breed in Poland, and the method of milking and processing milk obtained from them, in accordance with centuries-old shepherding traditions, has remained unchanged for centuries. Mountain sheep provide milk to produce traditional products (oscypek, bundz, bryndza, redykołka), wool, hides, and meat, which is appreciated by consumers both in Poland and abroad [10,18].

The Ministry of Agriculture and Rural Development in Poland maintains the List of Traditional Products which includes over two thousand items, 125 of which are cheeses and dairy products (https://www.gov.pl/web/rolnictwo/lista-produktow-tradycyjnych12, accessed on 1 March 2022). The List includes products whose unique features and properties or quality result from the application of traditional (used for at least 25 years) production methods. A product applying for inclusion in the List should be an element of the local community’s identity and belong to the cultural heritage of a specific region. The most numerous groups of products on the List are cheese made from goat’s milk and traditional products of mountain sheep farming. Some of them, such as the most recognizable regional cheese—Oscypek, as well as Redykołka and Bryndza Podhalańska (Table 2)—have been entered in the Register of Protected Designations of Origin (PDO), approved by the European Commission. Mountain sheep are an important part of the highland economy and culture. Sheep’s milk used in the production of these traditional sheep’s cheeses may come only from sheep of the Polish mountain sheep breed grazed in the specified geographical area. The allowable addition of cow’s milk from cows of the Polish Red breed may not exceed 40% of the total quantity of milk used to make these cheeses.

The meat product included in the List which has been granted Protected Geographical Indication (PGI) status is the Podhale lamb. The List of Traditional Products includes more items from native breeds of sheep. These are both dairy products, such as traditional żentyca; and diverse types of cheese, such as bundz, grudka, bryndza żywiecka and wołoska smoked cheese, wołoski smoked cheese and klagany cheese; as well as meat products, such as Beskidzka lamb, Świniarka lamb, lamb from Wielkopolska sheep, Jurassic lamb from Olkuska sheep, or sheep leg from Pomeranian sheep with garlic (https://www.gov.pl/web/rolnictwo/jagniecina-podhalanska, accessed on 1 March 2022).

The NRIAP has been carrying out numerous studies to analyse the composition and assess the quality of sheep products. Traditional dairy products such as bundz, oscypek or żentyca have many valuable and important components for human health, such as immunoglobulins, amino acids, or PUFA unsaturated fatty acids [19,20,21]. Meat obtained from lambs of native breeds, apart from its palatability, is characterised by a favourable fatty acid profile, PUFA n-6/n 3, and PUFA/SFA ratio [22].

#### 7.2.4. “Native Breed” Certification

**Figure 5:**
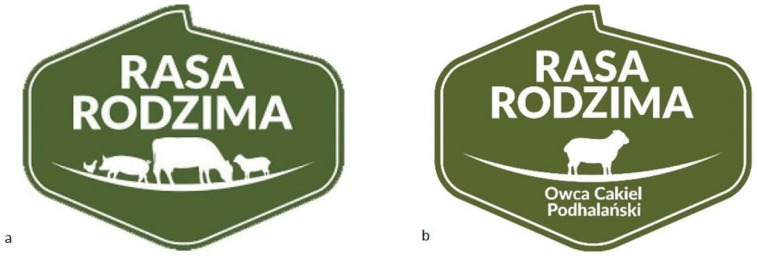
The NRIAP certification logo for Native Breed (**a**) and Native Breed—Podhale Zackel (**b**).

Considering the valuable properties of raw materials and products derived from local breeds, efforts have been made in many European countries to promote them through certification with unique quality marks. The NRIAP develops a certification system for native breeds and their products. As part of the project “Directions of use and protection of animal genetic resources under the conditions of sustainable development”, the Native Breed logo was developed as a collective symbol (Figure 5), as well as a separate one for each native breed, providing the basis for promotion of livestock native breeds as well as raw materials and products derived from them (http://ksb.izoo.krakow.pl/site/certification, accessed on 1 March 2022).

The logo system is not a quality system as defined by EU regulations, but it meets similar requirements. It is expected that the logo will promote the products of specific Polish native breeds kept under traditional (natural) systems on local markets. The main purpose of using the logo for producers of native breeds is to promote and raise public awareness of the role and importance of native breeds as an important part of our national heritage. This promotion is intended to highlight the importance of Polish native breeds, their historical as well as social and cultural value, the role they play in environmental and landscape renewal, and the potentials represented by their genetic resources. The logo is also intended to indicate products having a higher nutritional and health-promoting value. It may be used for all types of products (carcasses, meat and meat products, milk and milk products, eggs, honey, wool, hides, etc.) obtained from animals covered by the genetic resources protection programme. The logo is owned by the NRIAP, which establishes the principles and conditions for granting rights to its use. The right to mark products with the logo, based on an agreement with the Institute, belongs to breeders of domestic breeds participating in the programme, breeders-processors and processing plants producing products from the aforementioned breeds. The Institute maintains a register of participants in the certification programme, a website dedicated to it and an information campaign on the Native Breed logo [23].

#### 7.2.5. Farm Tourism and Other Forms of Local Entrepreneurship

**Figure 6:**
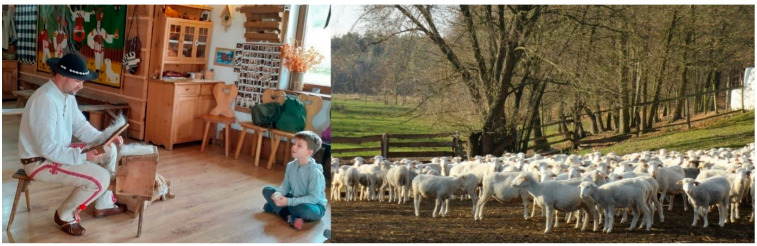
Education and agritourism farms breeding sheep of native breeds (https://ksp-zakopane.pl, accessed on 1 March 2022).

Keeping native breeds is popular among organic and farm-tourism farms, where they play an important popularising, promotional, and educational role not only for the animals themselves, but also for the region they come from (Figure 6). Such farms, often located within tourist-friendly regions with hiking and cycling trails, offer accommodation and facilities for many age groups, food produced by the farm, which is natural and minimally processed, as well as direct contact with animals and nature. Due to their good adaptation to local conditions, resistance to disease, healthiness and longevity, native breeds of animals are particularly useful in farm tourism, which is an essential element in protecting biodiversity [11,17]. An example of combining ecological and farm tourism activities is the certified “Nad Pilicą” Farm, located in the village of Krzętów in the Przedbork Landscape Park. The farm keeps sheep of the Świniarka breed, which is the native, most primitive breed of sheep in Poland. Visitors to the farm can learn about local customs and take part in activities and workshops on cultivating traditions, such as making cheese from sheep’s milk or sheep wool products—felting [24].

### 7.3. Increase in Public Awareness

**Figure 7:**
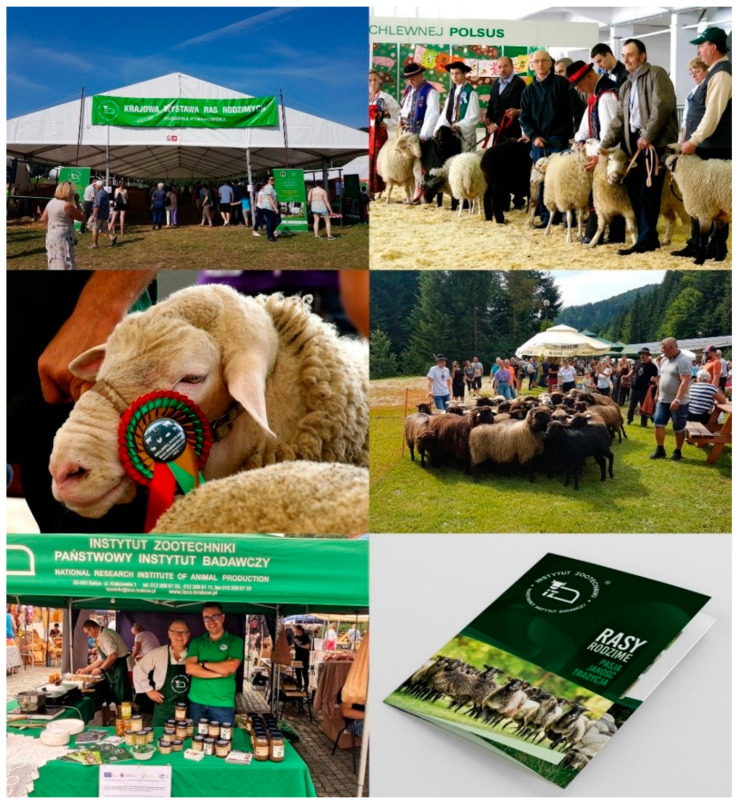
Exhibitions of native breeds organised by NRIAP and NRIAP promotional materials.

The dissemination of information on the benefits of preserving the animal’s genetic diversity and popularisation of the use of animal raw materials from domestic conservation breeds for the production of regional, organic products on small farms, gives breeders great opportunities to increase the profitability of their production. It may also constitute a form of income diversification. To this end, numerous promotional activities are undertaken, such as conferences, exhibitions, fairs, study tours, training courses, seminars, expert networks, and publications. An effective form of promotion of native breeds and products derived from them is animal exhibitions and thematic fairs and events, organised both as part of local and national events. The NRIAP holds regional and national exhibitions of native breeds in Poland, which are very popular among breeders and tourists. As shown in Figure 7, the main element of the exhibitions is the presentation of animals of species included in the genetic resources protection programmes, lectures by experts in the field of genetic resources protection, and the farm and environment programme, as well as managing the Institute’s information stand, where materials promoting the topic of native breeds (albums, leaflets, brochures, posters, promotional materials, film catalogues) are disseminated [25].

## 8. Vulnerability of Native Sheep Breeds

**Figure 8:**
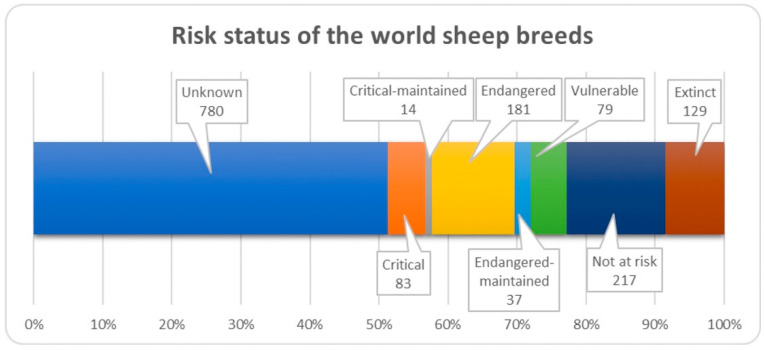
Risk status of the world sheep breeds.

Sheep, apart from cattle and horses, are the species with the largest number of endangered breeds (*n* = 181) (Figure 8); a large number of extinct sheep breeds have also been reported (*n* = 129). The species with the largest number of breeds (*n* = 183) reported as extinct are cattle, as well as pigs (*n* = 93) and horses (*n* = 92); some breeds may have become extinct before they were documented [3]. The status of many sheep breeds is unknown (*n* = 780), so it is important to continuously monitor the status of endangered species in order to implement appropriate conservation mechanisms to save valuable genotypes.

In Poland, and throughout the EU, in order to determine the endangered status of breeds until 2014, European Commission Regulation 1974/2006 was in force, setting out the criteria for the number of females within individual species [26]. It sets thresholds for the number of females below which a local breed is considered endangered. These thresholds, applicable in all EU countries, were set at 10,000 sheep. The initial studies defining the endangered status were proposed by Gandini [27], followed by Alderson [28] who defined the indicators of endangerment: numerical (number of females and effective population size), geographical (geographical concentration within the country), genetic (e.g., genetic erosion) and introgression (degree of crossbreeding). In addition to primary factors, anthropogenic, climatic, and epidemiological factors were also mentioned. European countries are now developing their own methods for determining risk status.

In Poland, a model based on three factors was proposed: demographic, genetic, and socio-economic [29]. Taking into account the results of international studies and the implementation of conservation programmes in Poland, a model was developed, including two main factors: the number of females (L), the effective population size (Ne) and the D-factor. A factor (D) was composed of six elements including: 1. geographical concentration; 2. demographic trend over the last 5 years; 3. cultural value; 4. origin control (DNA testing); 5. ex situ protection; 6. anthropogenic factors (existence of breeders’ associations, financial support, activity and age of breeders). According to the estimates, the majority of Polish native breeds are still considered endangered, and these require conservation actions, while the others require monitoring (Table 3).

## 9. Conclusions and Perspectives

The conservation of genetic resources of sheep breeding animals aims to maintain and increase the population size while striving to maintain the greatest possible genetic variability. The most effective method of conserving native breeds is their sustainable management. Keeping native breeds of animals on farms, in organic and tourism farms, as well as organising national and regional exhibitions, provides an opportunity to learn about and come into direct contact with the animals. Production, promotion, and marketing of quality products (using niche markets and short production chains) are essential to ensure the economic viability of farms. These activities must be accompanied by raising public awareness of indigenous breeds and their alternative use in environmental activities, as well as their role in preserving the cultural heritage of local communities, for example through mountain grazing and the production of traditional products. The certification of products from native breeds is a good form of direct promotion and a way of showing consumers the distinctiveness of local breeds and the high quality of the raw materials obtained from them. Certification increases consumer confidence in products derived from native breeds, and thus increases the interest in breeds themselves and the demand for these products. The endangered status of local breeds should be monitored and updated on an ongoing basis in order to respond to changes and to strengthen ongoing measures.

The programme for the protection of genetic resources of sheep is an important tool for the protection of biodiversity of this species, and its most visible effect is the constant growth of the protected population. Thanks to the implementation of the protection programme, after a long period of stagnation, sheep breeding is gaining more and more importance as one of the main field of livestock production in Poland.

## Figures and Tables

**Table 1:** Characteristics of Polish native breeds of sheep, divided into utility groups.

Breed	Population Size	% in the Program	Utility Type ^1^	Body Weight (kg)	Wool Type ^2^	Prolificacy (%)
Sheep with mixed wool						
Coloured Mountain Sheep (POG B)	2670	80.52	multi-purpose *	♂ 55 ♀ 45	coarse	125–130
Podhale Zackel (CP)	9434	84.40	multi-purpose	♂ 65 ♀ 45	coarse	125–130
Polish Mountain Sheep (POG)	550	62	multi-purpose	♂ 65 ♀ 45	coarse	125–130
Wrzosówka (WRZ)	9584	86.85	sheepskin	♂ 40 ♀ 32	coarse	≥150
Świniarka (ŚW)	2805	79.43	general-purpose	♂ 40–50 ♀25–35	coarse	120
Long-wool sheep						
Kamieniecka (KAM)	6437	87.93	dual-purpose **	♂ 90–110 ♀ 60–70	fine	140
Olkuska (OLK)	1520	77.96	prolificacy	♂ 50 ♀ 45	fine	≥ 180
Pomeranian (POM)	8946	91.99	dual-purpose	♂ 80–110 ♀ 65–75	fine	140
Polish Pogórza sheep (PG)	2090	74.59	dual-purpose	♂ 70–90 ♀ 55–60	fine	130
Lowland sheep						
Wielkopolska (WLKP)	9750	80.69	dual-purpose	♂ 100–120 ♀ 65–75	fine	140
Żelaźnieńska (ŻEL)	2500	78.84	dual-purpose	♂ 100–115 ♀ 55–65	fine	170
Corriedale (KOR)	2306	82.57	dual-purpose	♂ 100–130 ♀ 65–90	fine	130
Uhruska (UHR)	8441	88.67	dual-purpose	♂ 95–110 ♀ 55–80	fine	150
Merino sheep						
Old-type Polish Merino (MST)	9336	81.82	dual-purpose	♂ 90–100 ♀ 60–70	fine	≥ 125
Coloured Merino (MB)	926	85.21	dual-purpose	♂ 80–100 ♀ 55–65	fine	135
Meat sheep						
Black-headed (CZG)	4384	79.54	meat	♂ 90–110 ♀ 65–80	fine	120–140
White-headed meat sheep (BOM)	1200	70.33	meat	♂ 110–130 ♀ 70–80	fine	150

Explanations: ^1^ Utility type: * multi-purpose—milk, meat, skin, and wool; ** dual-purpose—wool and meat; ^2^ Wool type: fine—uniform, thin, and crossbred wool; coarse—thick and mixed.

**Table 2:** Traditional and regional products from Polish mountain sheep.

Product	Class	Data Registration in EU
Bryndza Podhalańska	1.3.—Cheeses	PDO, 12 June 2007
Oscypek	1.3.—Cheeses	PDO, 14 February 2008
Redykołka	1.3.—Cheeses	PDO, 1 December 2009
Jagnięcina Podhalańska	1.1. Fresh meat and offal	PGI, 9 October 2012

(Source: https://www.gov.pl/web/rolnictwo/produkty-zarejestrowane-jako-chronione-nazwy-pochodzenia-chronione-oznaczenia-geograficzne-oraz-gwarantowane-tradycyjne-specjalnosci, accessed on 1 March 2022).

**Table 3:** Risk status of the Polish national breeds of sheep.

Breed	Total Score (Points)	Risk Status
Pomeranian	2.6	EM *
Podhale Zackel	2.5	EM
Old-type Polish Merino	2.4	EM
Uhruska	2.3	EM
Wielkopolska	2.2	EM
Wrzosówka	2.2	EM
Żelaźnieńska	1.9	E **
Black-headed	1.9	E
Kamieniecka	1.9	E
Corriedale	1.8	E
Olkuska	1.8	E
Świniarka	1.8	E
Coloured Mountain Sheep	1.8	E
Polish Pogórza sheep	1.6	E
Coloured Merino	1.3	E

Explanation: the final result of the risk status assessment, assessed on a scale of 0 to 3 points, means: ≤1—critical hazard; >1 and ≤2—an endangered breed that requires protective measures; >2 and <3—an endangered breed that requires monitoring; ≥3—the breed is not endangered. * EM—endangered, requiring monitoring; ** E—endangered, requiring conservation.

## Data Availability

This review did not report any data.
